# Application of Machine Learning in the Prediction of the Acute Aortic Dissection Risk Complicated by Mesenteric Malperfusion Based on Initial Laboratory Results

**DOI:** 10.31083/RCM37827

**Published:** 2025-06-27

**Authors:** Zhechuan Jin, Jiale Dong, Jian Yang, Chengxiang Li, Zequan Li, Zhaofei Ye, Yuyu Li, Ping Li, Yulin Li, Zhili Ji

**Affiliations:** ^1^Department of General Surgery, Beijing Anzhen Hospital, Capital Medical University, 100029 Beijing, China; ^2^Department of Cardiovascular Surgery, Beijing Anzhen Hospital, Capital Medical University, 100029 Beijing, China; ^3^Beijing Institute of Heart, Lung and Blood Vessel Diseases, Beijing Anzhen Hospital, Capital Medical University, 100029 Beijing, China

**Keywords:** acute aortic dissection, mesenteric malperfusion, machine learning, prediction model

## Abstract

**Background:**

Mesenteric malperfusion (MMP) represents a severe complication of acute aortic dissection (AAD). Research on risk identification models for MMP is currently limited.

**Methods:**

Based on a retrospective study of medical records from the Beijing Anzhen Hospital spanning from January 2016 to June 2022, we included 435 patients with AAD and allocated their data to training and testing sets at a ratio of 7:3. Key preoperative predictive variables were identified through the least absolute shrinkage and selection operator (LASSO) regression. Subsequently, six machine learning algorithms were used to develop and validate an MMP risk identification model: logistic regression (LR), support vector classification (SVC), random forest (RF), extreme gradient boosting (XGBoost), naive Bayes (NB), and multilayer perceptron (MLP). To determine the optimal model, the performance of the model was evaluated using various metrics, including the area under the receiver operating characteristic curve (AUROC), accuracy, sensitivity, specificity, and the Brier score.

**Results:**

LASSO regression identified white blood cell count (WBC), neutrophil count (NE), lactate dehydrogenase (LDH), serum lactate levels, and arterial blood pH as key predictive variables. Among these, the WBC (OR 1.169, 95% confidence interval [CI] 1.086, 1.258; *p* < 0.001) and LDH levels (OR 1.001, 95% CI 1.000, 1.003; *p* = 0.008) were identified as independent risk factors for MMP. Among the six assessed machine learning algorithms, the RF model exhibited the best predictive capabilities, yielding AUROCs of 0.888 (95% CI 0.887, 0.889) and 0.797 (95% CI 0.794, 0.800) in the training and testing datasets, respectively, as well as sensitivities of 0.864 (95% CI 0.862, 0.867) and 0.811 (95% CI 0.806, 0.816), respectively, in the corresponding datasets.

**Conclusions:**

This study employed machine learning algorithms to develop a model capable of identifying MMP risk based on initial preoperative laboratory test results. This model can serve as a basis for making decisions in the treatment and diagnosis of MMP.

## 1. Introduction 

Acute aortic dissection (AAD) is a rapid onset, highly lethal cardiovascular emergency. Despite recent advancements in diagnostic and therapeutic techniques that have resulted in a reduction in hospital mortality, the overall prognosis remains poor [1]. Mesenteric malperfusion (MMP), a rare complication of AAD, is associated with the extension of the aortic dissection into the mesenteric arterial circulation. This can precipitate intestinal ischemia, necrosis, and a systemic inflammatory response, ultimately leading to multi-organ failure [2]. Previous studies have demonstrated that AAD patients with concomitant MMP have a significantly increased hospital mortality (13%–95%) [3, 4]. Given the subtle and non-specific early symptoms of MMP, delayed diagnosis frequently leads to irreversible intestinal necrosis, an independent risk factor that adversely impacts prognosis [5].

The clinical diagnosis of MMP currently relies on clinical symptoms, biochemical monitoring, and radiological studies [6]. However, most indicators undergo significant changes only after intestinal ischemia has progressed to an advanced stage, and the delay in diagnosis may result in missing the optimal window for intervention. Furthermore, the sensitivity and specificity of existing laboratory markers are limited [7], and the predictive capability of individual markers is insufficient to facilitate rapid risk stratification in emergency settings.

Machine learning has exhibited distinct benefits in cardiovascular medicine by integrating multidimensional data and analyzing nonlinear relationships [8]. However, its application in risk prediction for AAD combined with MMP is still in the exploratory stage.

This study combined patient demographic information with preoperative initial laboratory tests and used machine learning algorithms to create a risk identification model. The model is designed to address the constraints of traditional univariate analysis and achieve precise, individualized identification of the risk of MMP in patients with AAD. We compared the predictive capabilities of various algorithms to further elucidate the potential relationships between laboratory markers and MMP. The establishment of this model will facilitate the precise identification of patients at elevated risk for MMP, refine therapeutic approaches, and ultimately improve the outcomes of patients with AAD.

## 2. Materials and Methods

### 2.1 Study Population

**Fig. 1.**
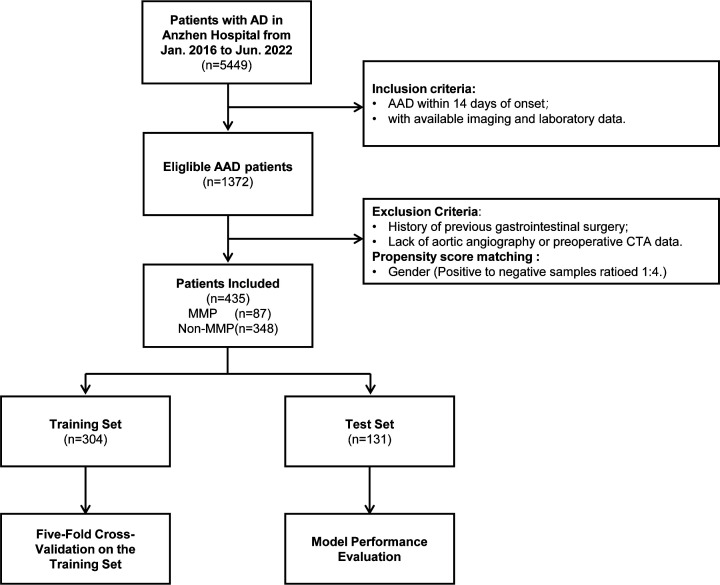
**Flowchart of the overall study**. AD, aortic dissection; AAD, acute aortic dissection; CTA, computed tomography angiography; MMP, mesenteric malperfusion.

This study is a single-center, retrospective observational study. We conducted a consecutive search of 5449 patients with aortic dissection who were treated at the Beijing Anzhen Hospital, Capital Medical University, from January 2016 to June 2022. Through a systematic screening process (see Fig. 1), 87 patients with MMP were ultimately selected. Due to the low incidence of MMP in AAD patients, and to avoid the issue of class imbalance during model training, we employed propensity score matching (PSM) and conducted gender-adjusted sampling of non-MMP patients at a 1:4 ratio (**Supplementary Table 1**). During the matching process, propensity scores were derived using Logistic regression. Subsequently, a negative event cohort comprising 348 patients was selected from a pool of 1263 non-MMP patients through nearest neighbor matching, employing a caliper width of 0.5 standard deviations, for the purpose of study analysis. The patients were divided into a training set (n = 304) and a testing set (n = 131) based on a 7:3 ratio, using randomly generated numbers.

The definitive diagnosis of MMP requires continuous observation and multi-dimensional evidence gathered during the patient’s clinical course [4, 9, 10]. Specifically, this includes the identification of images which confirm compression of the true lumen at or above the branch points of the mesenteric arteries (superior mesenteric artery or inferior mesenteric artery), or signs of mesenteric artery involvement by the dissection. Clinical manifestations, such as persistent abdominal pain, bloating, gastrointestinal bleeding, or peritoneal irritation signs, as well as laboratory abnormalities indicative of intestinal ischemia, including elevated serum lactate levels and increased white blood cell count (WBC) were also reviewed.

All study subjects met the following inclusion criteria: ① Diagnosis of AAD, as defined by the 2022 American College of Cardiology/American Heart Association guidelines (onset ≤14 days, including both Stanford A and B types) [11]; ② Availability of both radiology and laboratory data. Exclusion criteria focused on factors that may confound the assessment of intestinal perfusion, including the absence of preoperative computed tomography angiography (CTA) or aortic angiography, as well as a history of significant abdominal surgery.

The medical ethics committee of the Beijing Anzhen Hospital, Capital Medical University, approved this research protocol (approval number: KS2023020). Given the retrospective nature of this study, the requirement for obtaining patient consent was waived. The reporting of this study follows the strengthening the reporting of observational studies in epidemiology (STROBE) guidelines.

### 2.2 Data Collection

We extracted clinical data from the electronic medical record system of the patients, including demographic characteristics (such as age and gender), clinical manifestations (including gastrointestinal symptoms and ischemia of other organs), radiological data, and laboratory test results. The radiological data underwent a double-blind review via the hospital’s Picture Archiving and Communication System (PACS), with a focus on the Stanford classification of aortic dissection and the involvement of the superior and inferior mesenteric arteries. A total of 23 laboratory indicators were included, including routine blood tests, liver function, renal function, myocardial injury markers, coagulation function, as well as arterial blood pH and serum lactate levels. All collected laboratory tests were the first results obtained preoperatively within 12 hours of the patients’ initial presentation to ensure the timeliness of the data.

### 2.3 Machine Learning Model Construction

We initially addressed missing values through multiple imputation and conducted normalized preprocessing on all variables, including two demographic characteristics and 23 laboratory features. Subsequently, these variables were input into the least absolute shrinkage and selection operator (LASSO) regression to identify non-zero coefficient variables to develop the model. Multivariate logistic regression was then employed to assess the impact of these modeling features on the target event.

This study applied six representative supervised machine learning algorithms [12]: linear models (logistic regression [LR]), probabilistic inference (naive Bayes [NB]), ensemble learning (random forest [RF], extreme gradient boosting [XGBoost]), kernel methods (support vector classification [SVC]), and neural networks (multilayer perceptron [MLP]). Hyperparameter tuning for the models was performed using a grid search combined with five-fold cross-validation, allowing the identification of the optimal hyperparameter combinations for each algorithm within the training set (**Supplementary Table 2**).

We utilized a five-fold cross-validation technique to analyze the training set, with the objectives of evaluating the average performance and stability, and optimizing models. Subsequently, an independent test set was utilized to validate the final performance of the models. The area under the receiver operating characteristic curve (AUROC), accuracy, sensitivity and specificity were used to assess the classification discriminative ability of the model. The predicted probabilities’ accuracy was assessed using the Brier Score and calibration curve, while the clinical utility of the model was evaluated through Decision Curve Analysis (DCA). The Bootstrap method was adopted for 1000 resamplings to calculate the 95% confidence intervals for each indicator.

### 2.4 Interpretability Analysis of the Model

To elucidate the decision-making rationale of the machine learning models and enhance their clinical applicability, we employed SHapley Additive exPlanations (SHAP) for interpretability analysis. This method, based on cooperative game theory, calculates Shapley values to quantify the contribution of each feature to the current prediction, thereby explaining the importance of each variable [13].

### 2.5 Statistical Analysis

All statistical analyses were performed using SPSS (version 27.0, IBM SPSS Inc., Armonk, NY, USA). Categorical variables were presented as n (%), and analyzed using the chi-square test. Continuous variables with non-normal distribution were described as median and interquartile range [Median (P25, P75)], and statistical analysis was performed using the non-parametric rank-sum test. Statistical significance was defined as a two-sided *p* < 0.05.

## 3. Results

### 3.1 Patients Characteristics

A total of 435 AAD patients were included in the study, comprising 87 AAD patients with MMP. The baseline characteristics of the MMP and non-MMP groups are shown in Table 1. The majority of patients of the 400 patients were male (92%). Compared to the non-MMP group, patients in the MMP group were slightly younger (*p* = 0.042), more likely to present with abdominal symptoms (*p* < 0.001), and more prone to ischemia in other organs (*p* < 0.001). The in-hospital mortality rate was higher in the MMP group (*p* = 0.039). Additionally, laboratory results revealed that the MMP group had significantly higher WBC levels (*p* < 0.001), neutrophils (NE) (*p* < 0.001), alanine aminotransferase (ALT) (*p* = 0.002), aspartate aminotransferase (AST) (*p* < 0.001), blood urea nitrogen (BUN) (*p* < 0.001), creatinine (Cr) (*p* < 0.001), myoglobin (MB) (*p* < 0.001), creatine kinase MB (CK-Mb) (*p *= 0.012), lactate dehydrogenase (LDH) (*p* < 0.001), fibrin degradation products (FDP) (*p* < 0.001), D-dimer (*p* < 0.001), and serum lactate (*p* < 0.001) compared to the non-MMP group. The arterial blood pH levels (*p* = 0.007) in the MMP group showed a significantly lower trend.

**Table 1.** **Baseline characteristics of the non-MMP group and MMP group in AAD patients**.

Variables		Overall	Non-MMP	MMP	*p*-value
		(n = 435)	(n = 348)	(n = 87)	
Age, years		52.00 (42.00, 57.00)	52.00 (43.00, 57.00)	48.00 (41.00, 56.00)	0.042*
Male, (%)		400 (92%)	320 (92%)	80 (92%)	1.000
Type of AD, (%)					0.113
	Type A	208 (47.82%)	173 (49.71%)	35 (40.23%)	
	Type B	227 (52.18%)	175 (50.29%)	52 (59.77%)	
In-hospital mortality, (%)		44 (10.11%)	30 (8.62%)	14 (16.09%)	0.039*
Abdominal symptoms, (%)		136 (31.26%)	77 (22.13%)	59 (67.81%)	<0.001**
Other organs ischemia, (%)		130 (29.89%)	79 (22.7%)	51 (58.62%)	<0.001**
Mesenteric artery involvement, (%)		168 (38.62%)	83 (23.85%)	85 (97.7%)	<0.001**
WBC, (×10^9^)		11.39 (8.10, 14.86)	10.49 (7.74, 13.47)	15.51 (11.93, 18.18)	<0.001**
NE, (×10^9^)		9.39 (6.16, 12.83)	8.70 (5.52, 11.78)	13.35 (10.15, 16.46)	<0.001**
PLT, (×10^9^)		184.00 (148.00, 234.00)	187.00 (148.50, 243.00)	177.00 (148.00, 222.50)	0.123
Hb, (g/L)		143.00 (131.00, 155.00)	142.00 (131.50, 154.00)	147.00 (132.00, 155.50)	0.379
ALT, (U/L)		23.00 (15.00, 38.00)	22.00 (15.00, 33.50)	26.00 (19.00, 53.00)	0.002*
AST, (U/L)		21.00 (17.00, 33.00)	20.00 (16.00, 29.00)	31.00 (19.00, 57.00)	<0.001**
BUN, (mmol/L)		6.30 (5.10, 8.20)	6.07 (5.00, 8.00)	7.20 (5.65, 8.86)	<0.001**
Cr, (µmol/L)		80.90 (67.60, 103.00)	79.25 (67.30, 97.90)	92.40 (72.90, 134.50)	<0.001**
MB, (ng/mL)		37.10 (22.33, 73.90)	32.80 (20.60, 65.20)	59.60 (34.75, 151.75)	<0.001**
CK-MB, (ng/mL)		1.70 (1.00, 3.10)	1.60 (1.00, 2.90)	2.00 (1.30, 4.25)	0.012*
LDH, (U/L)		217.00 (177.00, 276.00)	210.50 (174.00, 260.00)	261.50 (206.00, 392.50)	<0.001**
Na, (mmol/L)		138.80 (136.60, 140.70)	138.80 (136.80, 140.60)	138.80 (135.60, 141.00)	0.336
K, (mmol/L)		3.87 (3.53, 4.20)	3.89 (3.54, 4.19)	3.85 (3.49, 4.23)	0.635
Tprotein, (g/L)		65.70 (61.65, 70.23)	65.90 (61.80, 70.15)	65.00 (61.20, 70.15)	0.556
Alb, (g/L)		39.35 (36.28, 42.70)	39.30 (36.25, 42.50)	39.60 (36.55, 43.00)	0.867
PT, (Second)		12.20 (11.40, 13.10)	12.20 (11.40, 13.00)	12.20 (11.45, 13.15)	0.783
APTT, (Second)		30.50 (27.90, 32.70)	30.50 (28.25, 32.80)	29.60 (27.10, 32.35)	0.089
INR, (%)		1.08 (1.02, 1.16)	1.08 (1.02, 1.16)	1.08 (1.01, 1.16)	0.524
FDP, (µmol/L)		12.59 (4.94, 36.54)	10.34 (4.07, 31.61)	20.30 (11.45, 48.40)	<0.001**
FBG, (g/L)		2.81 (2.10, 4.04)	2.90 (2.13, 4.17)	2.67 (2.04, 3.50)	0.065
D-Dimer, (mg/L)		1.21 (0.62, 2.94)	1.07 (0.51, 2.69)	2.03 (1.06, 3.96)	<0.001**
pH		7.42 (7.39, 7.45)	7.42 (7.40, 7.45)	7.41 (7.37, 7.43)	0.007*
Lactic, (mmol/L)		1.40 (1.00, 2.20)	1.35 (1.00, 2.00)	2.10 (1.10, 3.40)	<0.001**

Notes: Values are presented as n (%) or median (25th percentile, 75th percentile). *p < 0.05, **p < 0.001. Abbreviations: WBC, white blood cell count; NE, neutrophil count; PLT, platelet count; Hb, hemoglobin; ALT, alanine aminotransferase; AST, aspartate aminotransferase; BUN, blood urea nitrogen; Cr, creatinine; MB, myoglobin; CK-MB, creatine kinase-MB; LDH, lactate dehydrogenase; Na, sodium; K, potassium; Tprotein, total protein; Alb, albumin; PT, prothrombin time; APTT, activated partial thromboplastin time; INR, international normalized ratio; FDP, fibrin degradation products; FBG, fibrinogen.

### 3.2 Feature Selection and Risk Assessment

**Fig. 2.**
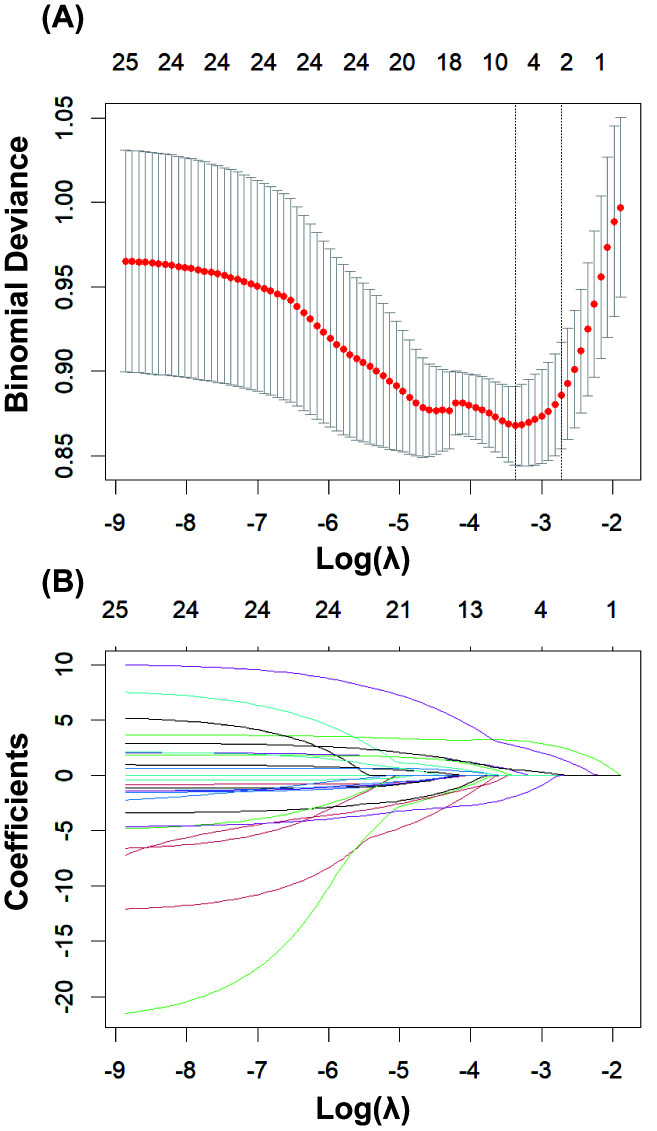
**LASSO regression for variable selection**. (A) Determining the optimal value for the regularization parameter (lambda). (B) Selecting 5 key predictor variables from 25 candidate variables using least absolute shrinkage and selection operator (LASSO) regression.

As shown in Fig. 2, LASSO regression was used to select the modeling features included in the model. By determining the optimal regularization coefficient (lambda), five key variables were selected from the 25 preoperative clinical variables, including WBC, NE, LDH, pH, and lactate. These five key variables were then fitted into a multivariate logistic regression analysis (Table 2). The results indicated that among the various predictive variables, WBC (odds ratio [OR] 1.169, 95% confidence interval [CI] 1.086, 1.258, *p* < 0.001) and LDH (OR 1.001, 95% CI 1.000, 1.003, *p* = 0.008) were independent risk factors for MMP, while pH was a protective factor. The remaining variables were identified as risk factors.

**Table 2.** **Multivariate Analysis of Predictive Factors for MMP**.

Variables	β	SE	Wald	OR (95% CI)	*p*-Value
WBC	0.156	0.037	17.487	1.169 (1.086, 1.258)	<0.001**
NE	0.022	0.018	1.628	1.023 (0.988, 1.058)	0.202
LDH	0.001	0.001	6.957	1.001 (1.000, 1.003)	0.008*
pH	–3.732	1.905	3.837	0.024 (0.001, 1.002)	0.050
Lactic	0.094	0.100	0.893	1.099 (0.904, 1.335)	0.345

Notes: *p < 0.05, **p < 0.001. The β coefficient, standard error, Wald value, and odds ratio for LDH have been rounded for presentation. Precise calculations reveal β = 0.001499, SE = 0.000568, Wald = 6.956747, OR = 1.001500 (95% CI: 1.000385, 1.002616), and the result is statistically significant (*p* = 0.008350). Abbreviations: SE, standard error; OR, odds ratio; CI, confidence interval.

### 3.3 Performance Analysis of Machine Learning Models

The five predictive features selected through LASSO regression were fitted into six machine learning algorithms. The results of the five-fold cross-validation analysis within the training set (as shown in Table 3) revealed that RF exhibited the optimal predictive performance, achieving an AUROC of 0.811 (0.756, 0.866) during validation. Conversely, the MLP of deep learning demonstrated the poorest performance, with an AUROC of 0.742 (0.670, 0.813).

**Table 3.** **AUROC Results from Five-Fold Cross-Validation on the Training Set**.

	Five-Fold Cross-Validation on the Training Set	
	n = 304	
Model name	Training Set AUROC (95% CI)	Validation Set AUROC (95% CI)
RF	0.924 (0.917, 0.930)	0.811 (0.756, 0.866)
NB	0.782 (0.765, 0.799)	0.763 (0.697, 0.830)
XGB	0.910 (0.901, 0.920)	0.785 (0.719, 0.851)
SVC	0.889 (0.875, 0.903)	0.745 (0.662, 0.827)
MLP	0.744 (0.729, 0.760)	0.742 (0.670, 0.813)
LR	0.785 (0.770, 0.800)	0.780 (0.717, 0.843)

Abbreviations: AUROC, area under the receiver operating characteristic curve; RF, random forest; NB, naive Bayes; XGB, XGBoost; SVC, support vector classification; MLP, multilayer perceptron; LR, logistic regression.

**Fig. 3.**
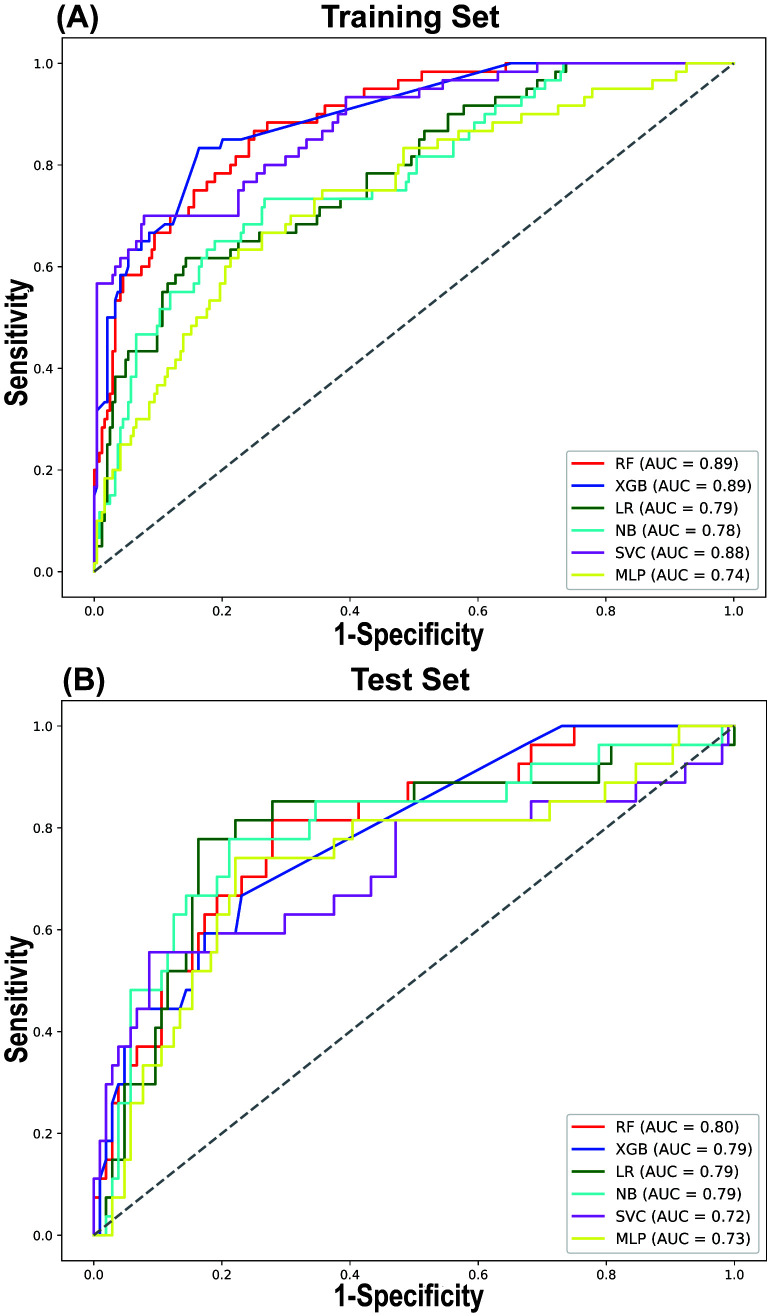
**LASSO regression for variable selection**. Comparison of receiver operating characteristic (ROC) curves for 6 different machine learning models in the Training Set (A) and Test Set (B). AUC, area under curve.

A comparative analysis of the predictive performance of each model in both the training and testing sets revealed consistent trends, with the RF model outperforming the others in terms of AUROC (Fig. 3). In the training and testing sets, the RF model demonstrated the best AUROC and sensitivity. The AUROC values were 0.888 (95% CI 0.887, 0.889) in the training set and 0.797 (95% CI 0.794, 0.800) in the testing set, while the sensitivity values were 0.864 (95% CI 0.862, 0.867) in the training set and 0.811 (95% CI 0.806, 0.816) in the testing set (Table 4). The SVC model exhibited the highest accuracy and specificity in the testing set, with values of 0.839 (95% CI 0.837, 0.841) and 0.914 (95% CI 0.912, 0.915), respectively (Table 4). However, its sensitivity was comparatively lower, at only 0.553 (95% CI 0.547, 0.559).

**Table 4.** **Performance of the Model on the Training and Test Sets**.

		AUROC (95% CI)	Accuracy (95% CI)	Sensitivity (95% CI)	Specificity (95% CI)	Brier (95% CI)
Training Set						
	RF	0.888 (0.887, 0.889)	0.773 (0.772, 0.775)	0.864 (0.862, 0.867)	0.751 (0.749, 0.753)	0.102 (0.101, 0.102)
	XGB	0.895 (0.894, 0.896)	0.837 (0.835, 0.838)	0.833 (0.830, 0.836)	0.837 (0.836, 0.839)	0.106 (0.106, 0.107)
	LR	0.786 (0.784, 0.788)	0.809 (0.808, 0.810)	0.616 (0.612, 0.620)	0.856 (0.855, 0.858)	0.128 (0.127, 0.129)
	NB	0.781 (0.778, 0.783)	0.736 (0.734, 0.737)	0.737 (0.733, 0.740)	0.735 (0.734, 0.737)	0.154 (0.152, 0.155)
	SVC	0.880 (0.878, 0.881)	0.879 (0.877, 0.880)	0.700 (0.696, 0.704)	0.923 (0.922, 0.924)	0.124 (0.123, 0.124)
	MLP	0.745 (0.743, 0.748)	0.747 (0.746, 0.749)	0.637 (0.633, 0.641)	0.774 (0.773, 0.776)	0.158 (0.157, 0.158)
Test Set						
	RF	0.797 (0.794, 0.800)	0.740 (0.738, 0.743)	0.811 (0.806, 0.816)	0.722 (0.719, 0.725)	0.133 (0.132, 0.135)
	XGB	0.786 (0.783, 0.789)	0.748 (0.746, 0.750)	0.669 (0.664, 0.675)	0.768 (0.766, 0.771)	0.137 (0.136, 0.138)
	LR	0.792 (0.789, 0.796)	0.824 (0.822, 0.826)	0.780 (0.775, 0.785)	0.836 (0.833, 0.838)	0.134 (0.133, 0.136)
	NB	0.792 (0.788, 0.795)	0.786 (0.783, 0.788)	0.776 (0.771, 0.781)	0.788 (0.786, 0.791)	0.157 (0.155, 0.158)
	SVC	0.715 (0.711, 0.719)	0.839 (0.837, 0.841)	0.553 (0.547, 0.559)	0.914 (0.912, 0.915)	0.144 (0.143, 0.146)
	MLP	0.727 (0.724, 0.731)	0.768 (0.766, 0.770)	0.741 (0.736, 0.746)	0.775 (0.773, 0.778)	0.165 (0.164, 0.166)

**Fig. 4.**
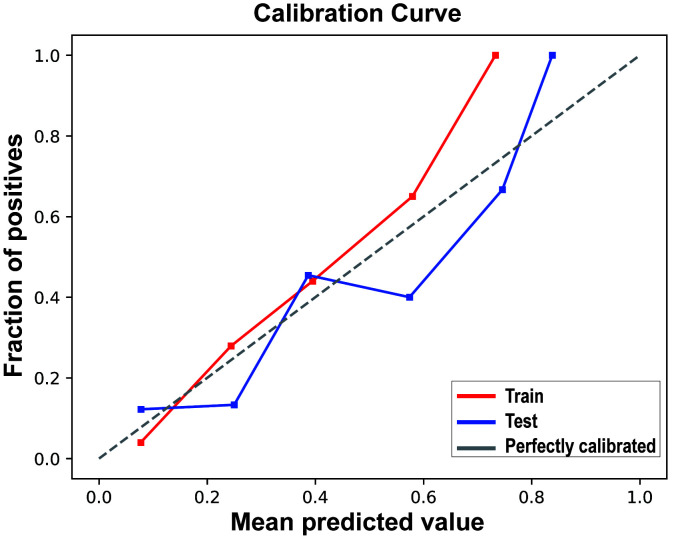
**Calibration curves of the random forest model in the Training Set and Test Set**.

**Fig. 5.**
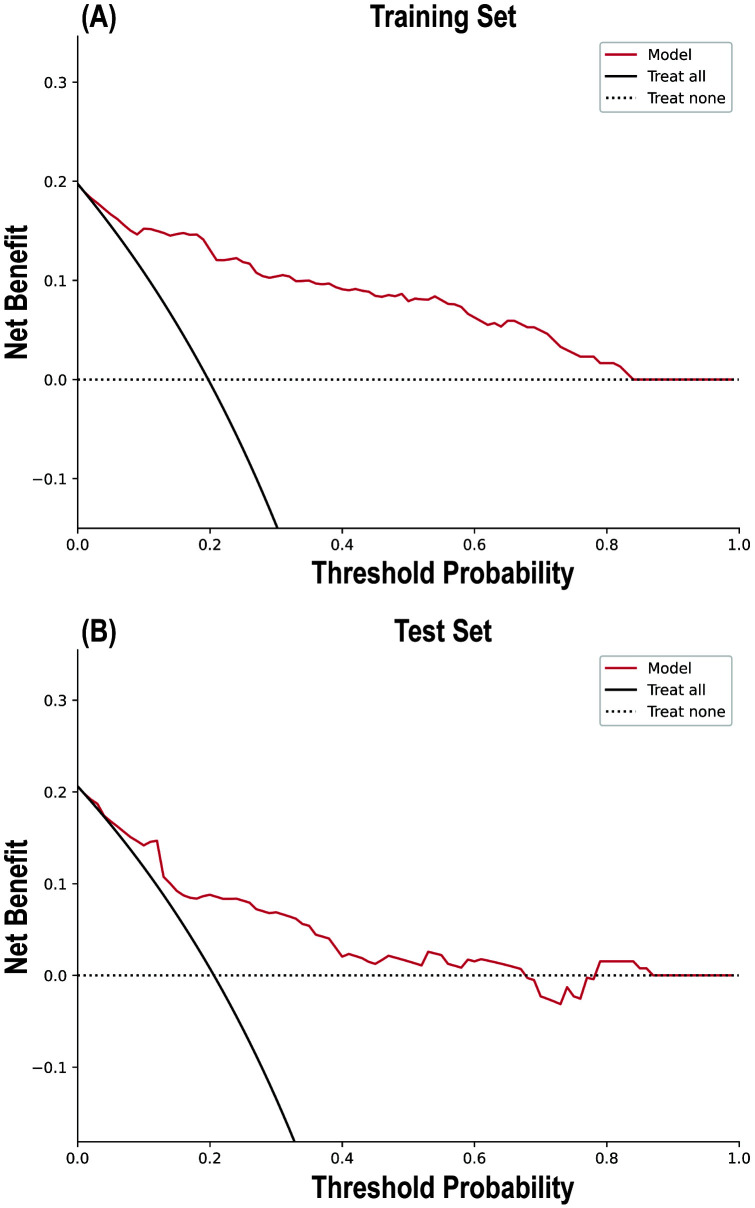
**Decision curve analysis of the random forest model in the Training Set (A) and Test Set (B)**.

The calibration curve results indicated good consistency between the predicted probabilities and actual observed frequencies for the RF model (Fig. 4), with Brier scores of 0.102 (95% CI 0.101, 0.102) and 0.133 (95% CI 0.132, 0.135) for the training and testing sets, respectively (Table 4). The DCA (Fig. 5) demonstrated that the RF model provided superior net benefits within a prediction probability range of 0 to 0.8, compared to extreme scenarios.

### 3.4 Interpretability of Machine Learning Models

**Fig. 6.**
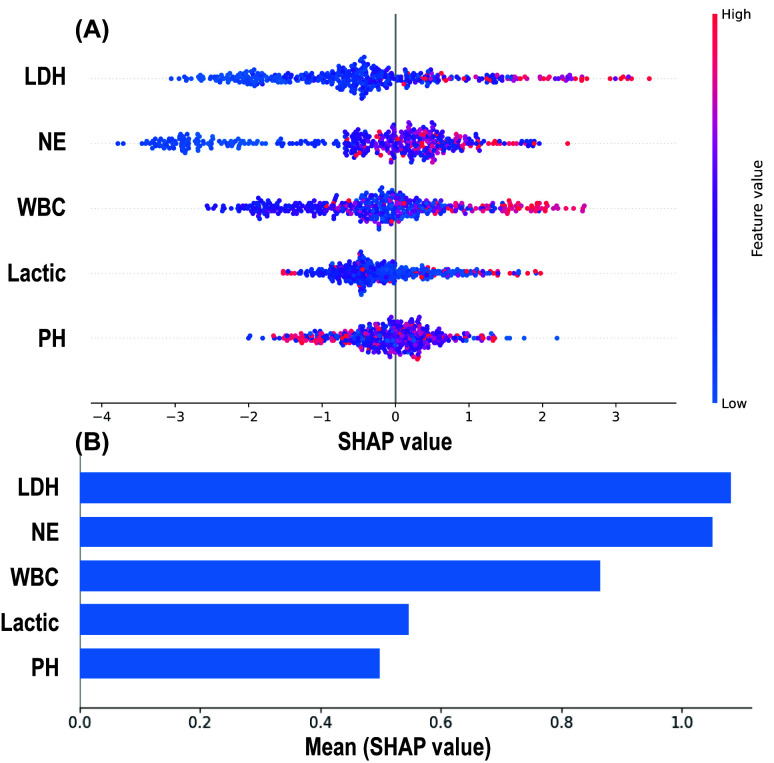
**SHAP Analysis Results of the Random Forest Model**. (A) SHAP summary plot: Each spot represents an individual patient, with red indicating high-risk values and blue indicating low-risk values. The x-axis represents the Shapley values. (B) Mean absolute Shapley values of 5 predictive variables. SHAP, SHapley additive exPlanations.

The SHAP algorithm was used to explain the RF model. By depicting a summary plot of the Shapley values, it was observed (Fig. 6A) that elevated levels of LDH, NE, WBC, and lactate were associated with a higher risk of MMP predicted by the model, while a decrease in pH was linked to an increased risk of MMP. Further comparison of the average absolute Shapley values for each predictive variable (Fig. 6B) revealed that LDH and NE had the greatest impact on the model’s predictions, while pH and lactate had the least influence.

## 4. Discussion

This study sought to enhance the ability to identify the risk of AAD complicated by MMP by developing and validating a machine learning-based prediction model. A total of 435 AAD patients were included in the study. Five preoperative laboratory test indicators, which were the initial results obtained after admission, were selected through Lasso regression. Various machine learning models were developed, with the RF model ultimately demonstrating the best performance in predicting the risk of MMP. Multivariate logistic regression analysis identified WBC (OR 1.169, 95% CI 1.086, 1.258, *p* < 0.001) and LDH (OR 1.001, 95% CI 1.000, 1.003, *p* = 0.008) as independent risk factors for concurrent MMP, while pH, NE, and lactate were determined to be non-independent predicting factors.

MMP is caused by dynamic obstruction resulting from compression of the true lumen by the false lumen of the dissection, or by static obstruction when the dissection extends into the mesenteric arteries. It is a condition induced by aortic dissection leading to acute mesenteric ischemia (AMI) [14]. The treatment of MMP presents several challenges. After repair of the aortic dissection, dynamic obstruction may improve; however, for static obstruction, restoring perfusion remains difficult. As a result, there is ongoing debate over whether to prioritize addressing the dissection or the perfusion deficit. Mortality rates vary widely depending on the dissection type and treatment strategy, ranging from 10% to 60% [3, 15].

Approximately 60% of patients with MMP present with gastrointestinal symptoms such as abdominal pain, bloating, gastrointestinal bleeding, and peritoneal irritation [4], consistent with the findings in this study (Table 1). However, these typical symptoms often appear late, usually indicating irreversible ischemic damage. Furthermore, MMP is more frequently associated with perfusion deficits in other organs, such as the lower limbs and kidneys [16, 17]. In this study, 58.62% of MMP patients had concurrent perfusion deficits in other organs.

Radiological examinations are crucial in the diagnosis of MMP [18]. Intestinal dilation, intestinal wall thickening or thinning, and reduced enhancement are signs of intestinal ischemia on gastrointestinal imaging studies [19]. However, due to the non-specific nature of these manifestations and their late onset, the sensitivity of early radiological imaging findings is only about 40% [20]. Lu *et al*. [9], identified that a decreased ratio of the true lumen to false lumen in the morphology of type B aortic dissection is an independent predictor of the risk for MMP. Radiological imaging is limited in providing dynamic assessments of the disease, making it challenging to detect MMP progression during dissection or to diagnose MMP caused by dynamic obstruction. Jonker *et al*. [21], reported that 20% of patients did not show abdominal vessel involvement on CTA.

Laboratory tests serve as a rapid assessment tool that is crucial for the diagnosis and real-time evaluation of MMP. In our study, we identified five key predictors associated with MMP risk, including elevated levels of LDH, NE, WBC, and lactate, as well as decreased pH values, all of which were correlated with an increased risk of MMP (Fig. 6). These findings are consistent with those reported in previous studies. It has been reported that intestinal mucosa is susceptible to damage under ischemic conditions, leading to disturbances in capillary blood flow and exacerbating local inflammatory responses, which in turn elevate inflammation [22]. Emile [23] found that an elevated WBC was closely related to the occurrence of intestinal necrosis (OR = 1.3, *p* < 0.0001), suggesting its potential as one of the predictive factors. Additionally, due to impaired tissue perfusion, metabolic acidosis may accompany intestinal ischemia and trigger abnormalities in biomarkers that reflect the degree of tissue perfusion. Khan *et al*. [7] reported that LDH and serum lactate levels were significantly elevated in patients with intestinal ischemia, with diagnostic AUROCs of 0.46–0.89 for LDH and 0.5–0.7 for serum lactate. Moreover, pH is also utilized to assess the severity of acidosis. Feier *et al*. [24] found that a preoperative pH ≤7.25 serves as an independent risk factor for the prognosis of patients with type A AAD complicated by organ malperfusion.

It is important to note that these indicators are usually elevated in all AAD patients, making it difficult to make the diagnosis of MMP. Blaser *et al*. [25], in a meta-analysis, reported that there is currently no single biomarker capable of accurately diagnosing mesenteric ischemia. Emerging biomarkers, such as intestinal fatty acid binding protein (I-FABP) and α-glutathione S-transferase (α-GST), are considered promising candidates [26], but their diagnostic value requires large-scale validation. Therefore, a comprehensive analysis and utilization of existing laboratory indicators are necessary for the diagnosis of MMP.

Machine learning has the ability to capture both linear and non-linear relationships within data, enabling better analysis of the intrinsic connections between target events and features. In recent years, machine learning-related research has gained popularity in the field of aortic dissection, covering various areas such as diagnosis, complications, and prognosis. Huo *et al*. [27] developed a diagnostic model for aortic dissection using 13 features and the NB model, achieving an AUROC of 0.81. Hata *et al*. [28] and Yi *et al*. [29] developed aortic dissection diagnostic models based on non-contrast-enhanced CT using convolutional neural networks, achieving AUROCs of 0.940 and 0.969, respectively. Dai *et al*. [30] utilized the XGBoost model to accurately predict acute kidney injury following aortic dissection repair, while Guo *et al*. [31] developed an in-hospital mortality prediction model for AAD using XGBoost.

However, there is a limited number of machine learning studies specifically focusing on MMP in patients with AAD. The majority of research has concentrated solely on AMI. Zhuang *et al*. [32] developed a logistic regression model utilizing variables such as WBC, BUN, neutrophil ratio, prothrombin time, and D-dimer, achieving an AUROC of 0.889 for predicting intestinal necrosis in AMI patients. While the variables included in this study are similar to ours, the performance of the model in the validation set was not reported, necessitating further validation. Additionally, Song *et al*. [33] developed a deep learning model that integrates CTA imaging features, albumin, and international normalized ratio, effectively identifying AMI patients from those suspected of an AMI with an AUROC of 0.96. These results suggest that deep learning methods may have a role in the detection and prognosis of MMP in AAD patients. Groesdonk *et al*. [34] created a diagnostic model for nonocclusive mesenteric ischemia based on clinical data from 865 patients undergoing elective cardiac surgery with extracorporeal circulation, using seven variables including postoperative serum lactate level, and achieved an accuracy rate of 93.9%. This study also focused on MMP as a cardiovascular complication, but in comparison, our study is more dedicated to the early and rapid identification of preoperative MMP risk in patients with AAD.

In this study, we constructed a total of six machine learning models to assess the risk of MMP. Among them, the RF model exhibited the best performance in the test set, achieving an AUROC of 0.797 (95% CI 0.794, 0.800), a sensitivity of 0.811 (95% CI 0.806, 0.816), and a specificity of 0.722 (95% CI 0.719, 0.725). In contrast, the XGBoost model demonstrated the highest AUROC in the training set, but there was a significant difference in performance between the training and test sets, suggesting the possibility of overfitting. To mitigate the risk of overfitting, we adopted measures such as class balancing, the introduction of regularization, and the restriction of model complexity. The results from the RF, NB, MLP, and LR models indicate that these measures were effective. However, excessive use of anti-overfitting measures may lead to the risk of underfitting, thus, further expansion of the sample size could be a highly effective method to enhance model performance. Additionally, the findings of our research reveal that RF outperforms XGBoost in overall performance, suggesting that RF may have advantages in small datasets and noisy data environments, which is consistent with the results of previous studies [35, 36, 37].

The primary objective of this study was to assess the risk of MMP based on preoperative laboratory test results obtained shortly after patient presented with an AAD. The results demonstrated that the RF model outperformed other machine learning models, achieving the highest AUROC of 0.797 in the test set. This model holds potential to provide personalized and accurate diagnostic and therapeutic decision-making data, thereby benefiting patients in clinical practice. Imaging tests remain critical in the diagnosis of MMP. Future research should explore the integration of deep learning and multimodal data approaches, incorporating more comprehensive information to construct more robust diagnostic prediction models.

### Limitations

This study has several limitations. First, the retrospective nature of the research introduces potential selection bias. Second, this study is limited to a single center, future studies should validate these findings in external cohorts. Additionally, the rarity of MMP results in a limited number of positive samples, and the use of undersampling techniques may potentially affect the generalization of the results. Finally, the study was confined to the initial laboratory test results obtained at the time of hospital admission, and other potentially important features were not included in the model.

## 5. Conclusions

This study found that WBC and LDH are high-risk factors for MMP. A machine learning model was developed, incorporating five preoperative laboratory test results, to identify high-risk populations for MMP, providing personalized and accurate information to support clinical decision-making.

## Availability of Data and Materials

The data regarding this article will be shared by the corresponding author upon reasonable request.

## Supplementary Material

Supplementary material associated with this article can be found, in the online version, at https://doi.org/10.31083/RCM37827.
