# Supplementary material for: Application of Machine Learning in the Prediction of the Acute Aortic Dissection Risk Complicated by Mesenteric Malperfusion Based on Initial Laboratory Results
Source: Rev Cardiovasc Med. 2025 Jun 27;26(6):37827. doi: 10.31083/RCM37827 (PMC12230842; doi:10.31083/RCM37827)
Supplement: Supplementary file 1 [file 2153-8174-26-6-37827-s1.zip › Supplementary Materials.docx]

Supplementary Materials

Contents

[Table 1. Propensity score matching result 2](#_Toc193725888)

[Table 2. Grid search hyperparameter tuning results 3](#_Toc193725889)

# Table 1. Propensity score matching result

|  | **Pre-PSM** | | **Post-PSM** | |
| --- | --- | --- | --- | --- |
|  | **MMP** | **Non-MMP** | **MMP** | **Non-MMP** |
| **Male** | 80(91.95%) | 975(77.20%) | 80(91.95%) | 320(91.95%) |
| **Famle** | 7(8.05%) | 288(22.80%) | 7(8.05%) | 28(8.05%) |

Abbreviations: PSM, propensity score matching; MMP, mesenteric malperfusion

# Table 2. Grid search hyperparameter tuning results

| **Model** | **Hyperparameter Grid Search**  **(Ranges)** | **Optimal Hyperparameters** | **Training Set AUROC (95%CI)** | **Validation Set**  **AUROC (95%CI)** | |
| --- | --- | --- | --- | --- | --- |
| **RF** | n_estimators (100, 110, 120, 130, 140) max_depth (2, 3, 4, 5) min_samples_split (2, 3, 4) min_samples_leaf (3, 4, 5) | n_estimators: 130 max_depth: 4  min_samples_split: 2 min_samples_leaf: 5 | 0.924(0.917, 0.930) | 0.811(0.756, 0.866) |  |
| **NB** | var_smoothing (10^-9, 10^-8, 10^-7, 10^-6, 10^-5, 10^-4, 10^-3, 10^-2, 10^-1, 1, 10^1, 10^2) | var_smoothing: 0.1 | 0.782(0.765, 0.799) | 0.763(0.697, 0.830) |  |
| **XGB** | n_estimators (5, 7, 9, 11) max_depth (1, 2, 3, 4) learning_rate (0.01, 0.06, 0.11, 0.16) gamma (0, 0.01, 0.02) min_child_weight (0, 1, 2, 3) | n_estimators: 9 max_depth: 3 learning_rate: 0.16 gamma: 0 min_child_weight: 2 | 0.91(0.901, 0.920) | 0.785(0.719, 0.851) |  |
| **SVC** | Cs (10^-8, 10^-7, 10^-6, 10^-5, 10^-4, 10^-3, 10^-2, 10^-1, 1, 10^1, 10^2, 10^3) kernel ('rbf', 'poly', 'linear', 'sigmoid') | Cs: 1000 kernel: rbf | 0.889(0.875, 0.903) | 0.745 (0.662, 0.827) |  |
| **MLP** | NA | Network Architecture:  3 Dense layers with 8, 32, and 16 neurons  Regularization: L2=0.01 Optimizer: Adam Learning Rate: 0.0001  Activation Function: ReLU, Sigmoid Training Epochs: 200 Dropout Rate: 50% | 0.744(0.729, 0.760) | 0.742(0.670, 0.813) |  |
| **LR** | C (0.001, 0.01, 0.1, 1, 10, 100) penalty ('L1', 'L2')  solver ('liblinear', 'saga') | C: 1 penalty: L1 solver: liblinear | 0.785(0.770, 0.800) | 0.780(0.717, 0.843) |  |

Abbreviations: AUROC, area under the receiver operating characteristic curve; CI, confidence interval; RF, random forest; NB, naive bayes; XGB, XGBoost; SVC, support vector classification; MLP, multilayer perceptron; LR, logistic regression.
